# The Role of Endocan Level in Determining Large and Small Vessel Occlusion in Acute Ischemic Stroke

**DOI:** 10.5152/eurasianjmed.2026.251094

**Published:** 2026-03-17

**Authors:** Erdal Tekin, Muhammet Çelik, Fatma Tortum, Ali Gür, İbrahim Özlü, Mehmet Nuri Koçak, Özcan Ağyürek, Mustafa Bayraktar

**Affiliations:** 1 Department of Emergency Medicine, Atatürk University Faculty of Medicine, Erzurum, Türkiye; 2 Department of Medical Biochemistry, Atatürk University Faculty of Medicine, Erzurum, Türkiye; 3 Department of Neurology, Atatürk University Faculty of Medicine, Erzurum, Türkiye; 4 Department of Family Medicine, Atatürk University Faculty of Medicine, Erzurum, Türkiye

**Keywords:** Biomarker, endocan, ischemic stroke, large vessel occlusion, stroke

## Abstract

**Background:**

Stroke is a clinical condition in which blood flow to a brain area is interrupted by occlusion or hemorrhage. At present, the diagnosis and treatment of stroke are based on the use of biochemical markers and imaging methods. The degree of inflammation in the vessel wall may be reflected by changes in endocan levels. The aim of this study was the investigation of the role of endocan blood levels in the determination of large- and small-vessel occlusion in acute ischemic stroke.

**Methods:**

This was a prospective, single-center clinical study. Sociodemographic characteristics, physical examination, laboratory findings, and endocan levels were recorded. Patients with ischemic stroke were categorized as large-vessel occlusive (LVO) or small-vessel occlusive (SVO) on computed tomography (CT) angiography. Blood endocan levels were compared between the 2 groups.

**Results:**

A total of 110 patients were included in the study; the mean age of these patients was 69.2 years and 50.5% were male. Large-vessel occlusive was present in 33.3% (n = 31) of these patients, and endocan levels were higher in the LVO group but without statistical significance (*P* > .05). Modified Rankin Score was higher in the LVO group while Glasgow Coma Score was lower and both were statistically significant (*P* < .001). The area under the receiver-operating characteristic (ROC) curve for troponin was 0.762 and statistically significant (*P* = .001).

**Conclusion:**

Serum endocan levels did not differ significantly between LVO patients and controls. However, more research is needed to determine how significant these high endocan levels are in diagnosing major artery disease.

**Main Points**

- Stroke is a clinical condition in which blood flow to a brain area is interrupted by occlusion or hemorrhage.
- Large vessel occlusion (LVO) represents a critical form of ischemic stroke, resulting from the acute blockage of a major intracranial artery, leading to extensive cerebral infarction and a high probability of poor functional outcome.
- Endokan is an inflammation marker that indicates endothelial dysfunction and may be helpful in diagnosing stroke patients.
- In the study, serum endocan levels were found to be higher in patients who had a stroke due to large vessel occlusion.

## Introduction

Stroke, a condition resulting from a lack of blood flow to a part of the brain due to occlusion or hemorrhage, is a leading cause of disability and death worldwide. Ischemic stroke accounts for 87% of all strokes and is usually caused by a large vessel occlusion (LVO).^1^^,^^2^ In particular, large vessel ischemic stroke poses a high risk for patients requiring emergency intervention. Therefore, the diagnosis of large vessel ischemic stroke should be accurate and rapid. Currently, biochemical markers and imaging techniques are used in stroke diagnosis and treatment planning.^3^ Several biochemical markers have been studied and one of these potential biochemical markers is endocan.

Endocan, which is a proteoglycan, has been accepted as an indicator of angiogenesis and endothelial cell activation. Significant associations have been reported between elevated endocan levels and various inflammatory, cardiovascular, and cerebral ischemic diseases. All these findings suggest that changes in endocan levels may reflect the degree of inflammation in the vessel wall.^4^^,^^5^ As a possible cause of ischemic stroke is atherosclerotic-inflammatory vascular involvement, it can be argued that elevated serum levels of endocan in ischemic stroke may indicate large-vessel occlusion. With this in mind, the aim of this study was to investigate the blood levels of endocan in the determination of large- and small-vessel occlusion in acute ischemic stroke.

## Material and Methods

This study is a prospective, single-center clinical research. The study was conducted at Atatürk University, Faculty of Medicine, Health Research and Application Center. This study was approved by the Ethics Committee of Atatürk (approval number: 04-26, date: April 28, 2022). Patients and/or their relatives were informed about the study, and informed consent was obtained. Helsinki Declaration criteria were taken into consideration during the study. The study was supported by Atatürk University BAP unit with the ID number 10922 and project code number TSA-2022-10922.

Patients between the ages of 18-85 years, those who voluntarily agreed to participate in the study, patients who had acute ischemic stroke for the first time, and patients without a history of stroke were included in the study. Patients younger than 18 years and older than 85 years, patients who refused to participate in the study, pregnant and lactating patients, patients who could not undergo computed tomography (CT) angiography or for whom CT angiography was not indicated, patients with hemorrhagic or traumatic stroke, patients with a history of previous stroke, patients with malignancy, chronic liver disease, hematologic disease, autoimmune disease, and acute or chronic inflammatory disease were excluded.

Patients admitted to the emergency department were evaluated for eligibility for the study, and vital signs, Modified Rankin Scale, and Glasgow Coma Scale (GCS) scores were recorded at the time of initial presentation. The CT/CT angiography was performed to differentiate ischemic and hemorrhagic stroke. In addition, a blood sample was taken for endocan level during routine blood sampling, and the blood taken for endocan level was centrifuged and stored at −80°C. Demographic data including age, gender, past medical history, and smoking history were recorded. Biochemical parameters including blood glucose, lipid levels, creatinine, homocysteine, homocysteine, procalcitonin, and high-sensitivity C-reactive protein were recorded.

According to the findings of CT angiography, ischemic stroke patients were divided into 2 groups: large vessel occlusion (LVO) and small vessel occlusion (SVO). The discharge or death status of the patients was recorded, and the discharged patients were contacted and the Modified Rankin Scale was checked 3 months later.

### Statistical Analysis

The data obtained were statistically analyzed using SPSS 23.0 software (IBM, USA). Normally distributed numerical data are presented as mean and standard deviation, non-normally distributed data as median (25-75); categorical data are presented as frequency and percentage. The normal distribution of the data was evaluated by Shapiro–Wilk test. In the analysis of numerical data, Student’s *t-*test was used if there was a normal distribution and Mann–Whitney *U-*test was used if there was no normal distribution. Categorical data were compared with the chi-square test. A receiver-operating characteristic (ROC) curve analysis was performed to determine mortality. A value of *P* < .05 was taken for significance in all studies.

## Results

The study included 110 patients. The mean age of these patients was 69.2 years and 50.5% were male. Of these patients, 33.3% (n = 31) had LVO. Of the patients with LVO, 11 (35.5%) were male and the mean age was 68.8 years. There was no statistical significance between both groups (*P* > .05). There was no statistical significance between the prehospital stroke scale (FAST) and vital signs of the groups (*P* > .05). When the treatments administered to the groups were analyzed, 10 (32.3%) of the patients in the LVO group received antiaggregant+anticoagulant therapy, while 46 (74.2%) of the patients in the non-LVO group received antiaggregant + anticoagulant therapy, which was statistically significant (*P* < .001). Sociodemographic, clinical, and anamnestic data of the patients are given in Table 1.

When the laboratory data of the patients were compared, cholesterol, triglyceride, low-density lipoprotein, high-density lipoprotein, creatinine, lactate, procalcitonin, homocysteine, vitamin D, ferritin, and endocan levels were higher in the LVO group but not statistically significant (*P* > .05). Modified Rankin score was higher and Glasgow coma score was lower in the LVO group, and both values were statistically significant (*P* < .001). The laboratory parameters, Modified Rankin score, and Glasgow Coma Score of the patients are given in Table 2.

**Figure 1.**
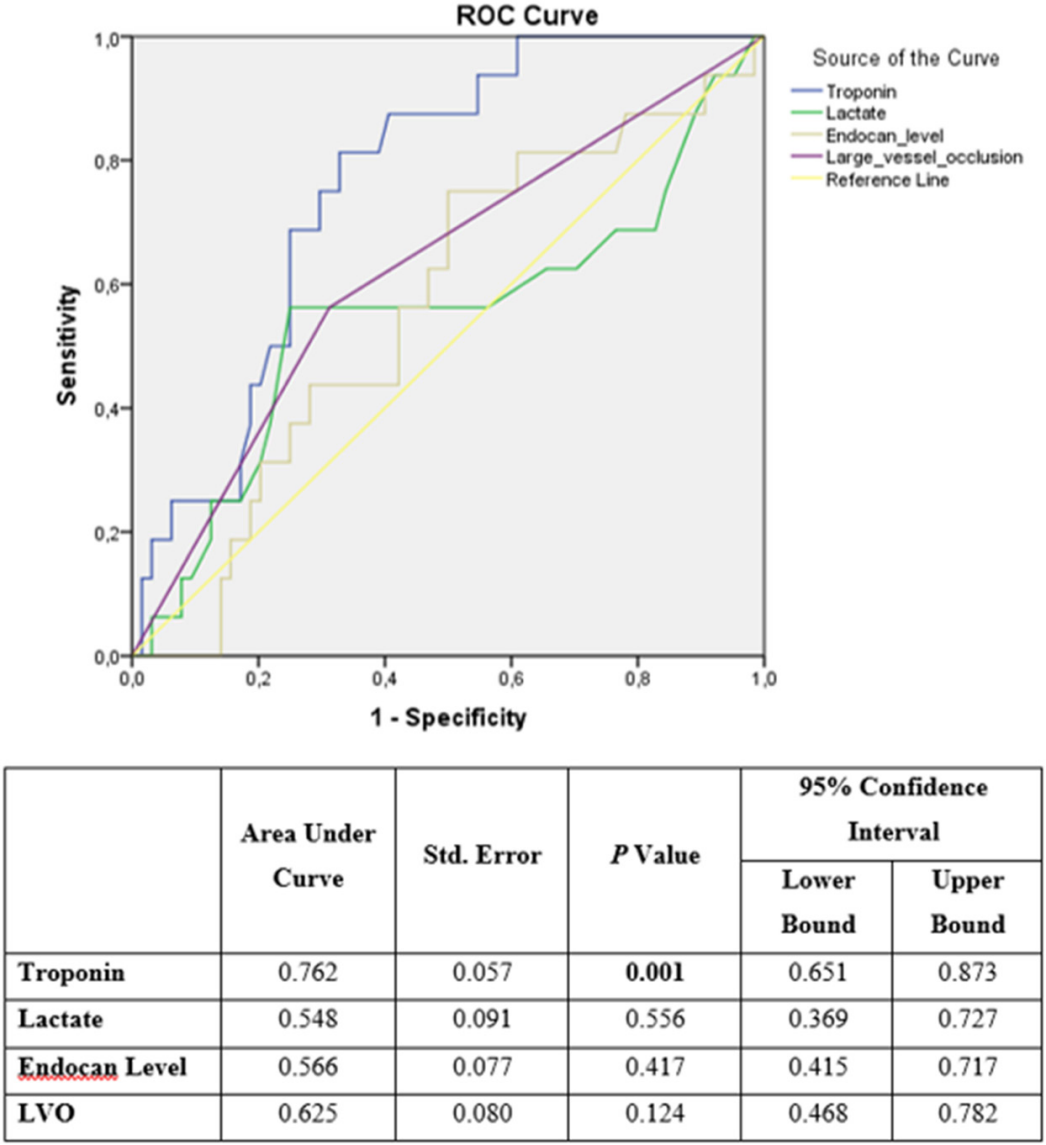
ROC curve of troponin, lactate, endocan level and large vessel occlusion.

Prediction of mortality by troponin, lactate, endocan level and major vessel involvement is shown in Figure 1. Accordingly, the area under the ROC curve of troponin value was 0.762 and statistically significant (*P* = .001). The AUC value of lactate, endocan and major vascular pathology was low and not statistically significant (*P* > .05) (Figure 1).

## Discussion

The study is a prospective study investigating the role of serum endocan levels in determining LVO in acute ischemic stroke. Although endocan levels were high in patients with LVO, its role in LVO was not statistically significant. Also, its role in determining mortality was not statistically significant. However, He XW et al emphasized that the level of endocan was higher in patients with LVO stroke and may be helpful in predicting short-term adverse outcomes.^6^ In the literature, it has been emphasized that endocan increases the production of proinflammatory cytokines by endothelial cells, increases microvascular permeability and regulates the aggregation, adhesion, and subsequent migration of leukocytes along the endothelium.^7^^,^^8^ In addition, Menon P et al reported that increased expression of endocan was detected in atherosclerotic lesions.^9^ Increased serum endocan levels have been observed in association with the presence and/or severity of atherosclerosis-related diseases such as coronary artery disease and ST elevation myocardial infarction.^10^^,^^11^

He XW et al took endocan blood samples in the early period in their study on ischemic stroke patients and emphasized that serum endocan levels were higher in LAA stroke patients.^6^ However, Rocha SF et al found that endocan levels increased 24 hours after induction of cerebral ischemic-reperfusion injury in their study on mice.^12^ In this study, blood endocan samples were obtained at the time the patients were admitted to the emergency department. Therefore, there were differences in blood endocan sampling times in the study.

Endocan is a marker of endothelial dysfunction because it plays a central role in pathologic events of the vascular endothelium.^13^ Therefore, endocan levels were aimed to investigate in ischemic stroke in which vascular endothelial damage plays a role. In the literature, Balta et al showed that endocan levels were significantly higher in Behçet’s disease characterized by vascular inflammation. In the same study, it was emphasized that circulating endocan levels may be a marker of Behçet’s disease activity. It was also reported that endocan levels were positively correlated with cardiovascular risk and disease activity in these patients.^14^^,^^15^ Balta S et al emphasized that endocan is a new marker in endothelial functions.^4^ They emphasized that endocan blood levels are a marker in inflammations, malignancies, atherosclerosis, hypertension, peripheral arterial diseases, obesity, metabolic syndromes, and sepsis and are strongly associated with these.^4^^,^^16^ In this study, vascular endothelial damage was also present in stroke patients, but it was thought that LVO was not statistically significant in stroke because stroke was accompanied by other chronic diseases. In future studies, it is believed that more reliable results will be obtained if stroke patients without chronic vascular endothelial damage are included in the study.

### Limitations

The study has several limitations that should be considered. First, blood samples for serum endocan measurement were obtained upon admission to the emergency department. Since the time elapsed between the onset of stroke symptoms and hospital admission varied among patients, the timing of sample collection could not be standardized. Given that endocan levels may exhibit temporal fluctuations following an acute ischemic event, this lack of synchronization might have influenced the observed serum concentrations and their statistical significance. Second, the single-center design and relatively small sample size may limit the generalizability and statistical power of the results. Third, endocan levels were only measured at admission, preventing the assessment of temporal changes. Lastly, despite strict exclusion criteria, the potential impact of unmeasured comorbidities on endocan levels cannot be entirely ruled out.

## Conclusion

In conclusion, no statistically significant difference was observed in endocan serum levels between LVO stroke patients and the control group. Despite the high endocan levels, the potential role of these findings in predicting or identifying large vessel occlusion requires further detailed studies.

Externally peer-reviewed.

## Figures and Tables

**Table 1.** Sociodemographic, Clinical, and Anamnestic Data of the Patients

**Variables**	**Total ** **N = 93 (100%)**	**LVO ** **N = 31 (33.3%)**	**SVO ** **N = 62 (66.7%)**	*P*
Men, n (%)	47 (50.5)	11 (35.5)	36 (58.1)	.686
Age, mean ± SD	69.2 ± 16.8	68.8 ± 17.7	69.4 ± 16.4	.990
Smoking, n (%)	35 (37.6)	11 (35.5)	24 (38.7)	.763
Hypertension, n (%)	65 (69.9)	24 (77.4)	41 (66.1)	.266
Diabetes mellitus, n (%)	35 (37.6)	11 (35.5)	24 (38.7)	.763
COPD, n (%)	10 (10.8)	3 (9.7)	7 (11.3)	.814
CRF, n (%)	3 (3.2)	0 (0.0)	3 (4.8)	.216
CAD, n (%)	42 (45.2)	20 (64.5)	22 (35.5)	**.008**
Dyslipidemia, n (%)	12 (12.9)	6 (19.4)	6 (9.7)	.192
Stroke, n (%)	13 (14.0)	5 (16.1)	8 (12.9)	.674
Prehospital stroke scale (FAST) Face, n (%) Arm, n (%) Speech, n (%) Time, hour, median (Q25; Q75)	31 (33.3) 70 (75.3) 39 (41.9) 2.00 (1.00-5.50)	8 (25.8) 25 (80.6) 11 (35.5) 2.00 (1.00-3.00)	23 (37.1) 45 (72.6) 28 (45.2) 2.65 (1.38-6.00)	.279 .398 .375 .369
SBP, mmHg, median (Q25; Q75)	150.0 (140.0-172.5)	148.0 (140.0-170.0)	153.0 (139.8-176.0)	.324
DBP, mmHg, median (Q25; Q75)	89.0 (80.0-96.9)	89.0 (84.0-98.0)	87.5 (80.0-95.5)	.665
Heart rate, min, median (Q25; Q75)	82.0 (73.5-91.5)	86.0 (79.0-92.0)	79.0 (71.8-90.3)	.076
Respiratory rate, min, median (Q25; Q75)	15.0 (14-16)	15.0 (14.0-16.0)	14.5 (14.0-16.0)	.909
Body temperature, °C, median (Q25; Q75)	36.5 (36.4-36.6)	36.5 (36.4-36.6)	36.5 (36.4-36.7)	.663
Oxygen saturation, %, median (Q25; Q75)	92.0 (88.5-96.0)	90.0 (89.0-94.0)	93.0 (88.0-96.0)	.141
CT finding, yes, n (%)	26 (28.0%)	10 (32.3)	16 (25.8)	.516
Treatment, n (%) Antiaggregant +Anticoagulant Thrombolytic Thrombectomy	56 (60.2) 24 (25.8) 13 (14.0)	10 (32.3) 8 (25.8) 13 (41.9)	46 (74.2) 16 (25.8) 0 (0)	**<.001**
Quarterly outcome, ex, n (%)	18 (19.4)	9 (29.0)	9 (14.5)	.097

CAD, coronary artery disease; COPD, chronic obstructive pulmonary disease; CRF, chronic renal failure; CT, computed tomography; DBP, diastolic blood pressure; LVO, large vessel occlusion; SBP, systolic blood pressure; SVO, small vessel occlusion.

**Table 2.** Patients’ Laboratory Parameters and Modified Rankin Score and Glasgow Coma Score

**Variables, Median (Q25; Q75)**	**Total ** **N = 93 (100%)**	**LVO ** **N = 31 (33.3%)**	**SVO ** **N = 62 (66.7%)**	***P***
Glucose, mg/dl mean ± SD	129.8 ± 64.5	120.4 ± 56.9	134.7 ± 67.9	.271
Cholesterol, mmol/L	177.3 (137.4-216.8)	183.4 (138.0-220.1)	174.3 (136.6-214.7)	.687
Triglycerides, mmol/L	114.5 (65.8-135.0)	116.8 (57.0-144.0)	113.3 (68.8-133.5)	.507
LDL-C, mmol/L	101.9 (71.2-125.1)	109.5 (72.1-137.0)	98.2 (69.1-121.2)	.275
HDL-C, mmol/L	47.2 (37.8-53.8)	48.1 (42.1-53.1)	46.7 (36.6-54.6)	.507
Creatine, mg/dl	0.97 (0.65-1.06)	1.11 (0.64-1.14)	0.91 (0.65-1.04)	.549
Lactate, mmol/L	2.35 (1.45-2.70)	2.55 (1.40-2.70)	2.25 (1.50-2.75)	.546
hs-cTnI, ng/L	54.2 (4.4-23.8)	19.5 (4.5-22.5)	73.9 (4.2-24.2)	.912
C-reactive protein, mg/L	29.3 (4.4-40.1)	28.6 (6.4-36.2)	29.7 (3.9-41.8)	.429
Procalcitonin, µg/L	0.6 (0.1-0.9)	0.8 (0.1-1.4)	0.5 (0.2-0.7)	.629
Homocysteine, μmol/L	16.8 (10.4-19.5)	17.3 (11.1-20.5)	16.5 (9.7-18.8)	.379
Folate, ng/mL	9.8 (6.9-11.2)	9.7 (6.4-11.6)	9.8 (7.4-10.9)	.851
Vitamin B12, pg/mL	421.9 (201.0-373.5)	357.4 (194.0-360.0)	454.1 (201.0-379.8)	.744
Vitamin D, nmol/L	22.7 (5.5-22.0)	40.9 (6.7-25.6)	13.6 (5.2-17.8)	.145
Ferritin, ng/mL	139.6 (42.3-129.2)	159.4 (51.6-1137.0)	129.7 (32.4-122.9)	.399
Endocan level, pg/mL, mean ± SD	380.4 ± 253.8	477.3 ± 348.4	347.1 ± 184.5	.383
Modified Rankin Score	3.59 (2.0-5.0)	4.39 (3.0-6.0)	3.19 (2.0-5.0)	**.002**
GCS	13.6 (13.0-15.0)	12.6 (11.0-15.0)	14.2 (14.8-15.0)	**.008**

GCS, Glasgow Coma Score; HDL, high-density lipoprotein cholesterol; LDL-C, low-density lipoprotein cholesterol; LVO, large vessel occlusion; SVO, small vessel occlusion; TnI, troponin-I.

## Data Availability

The data that support the findings of this study are available on request from the corresponding author.
